# Interparental conflict, not divorce, is linked to less positive marital attitudes among Chinese emerging adults

**DOI:** 10.1186/s40359-026-04232-y

**Published:** 2026-02-24

**Authors:** Honghong Xu, Yingjuan He

**Affiliations:** 1 Medical Psychology Department, School of Health Humanities, Peking University, No. 38 Xueyuan Road, Haidian District, Beijing, 100191 China; 2 School of Public Health, Mudanjiang Medical University, Mudanjiang, 157011 China

**Keywords:** attachment avoidance, marital attitudes, interparental conflict, meaning in life, emerging adulthood, China

## Abstract

**Background:**

Interparental relationship quality and attachment are theorized to be associated with young adults’ marital attitudes, yet evidence in Chinese cultural contexts is mixed. We tested whether interparental conflict versus divorce differentially relates to marital attitudes and whether meaning in life adds explanatory value.

**Methods:**

Participants were 398 Chinese university students (mean age = 20.8 years; 40.6% male) who completed measures of parental marital status/conflict (harmonious; high conflict without divorce; divorced; bereaved), adult attachment, meaning in life, and marital attitudes (higher scores = more positive). We reported descriptives and correlations, then ran hierarchical regressions and tested mediation/moderation by meaning in life.

**Results:**

Attachment avoidance was negatively associated with marital attitudes, whereas meaning in life was positively associated. Men reported more positive marital attitudes than women. Compared with respondents from harmonious intact families, those from high-conflict (non-divorced) families reported more negative marital attitudes; no difference emerged between divorced and harmonious families. Mediation and moderation tests showed that meaning in life neither explained nor buffered the association between avoidance and marital attitudes.

**Conclusions:**

Findings underscore the salience of interparental conflict—rather than divorce per se—and attachment avoidance in predicting Chinese emerging adults’ marital attitudes. Meaning in life showed concurrent positive links with attitudes but no indirect or buffering role.

**Supplementary Information:**

The online version contains supplementary material available at 10.1186/s40359-026-04232-y.

## Introduction

The landscape of marriage and family is changing rapidly in contemporary China, particularly among young people transitioning to adulthood. As emerging adults navigate accelerated socioeconomic development, expanding educational opportunities, and evolving cultural norms, traditional marital attitudes are being reconsidered [1, 2]. Because beliefs formed during this developmental window have downstream implications for relationship formation and stability, it is important to clarify the psychological processes linked to marital attitudes in this population.

Attachment theory offers a powerful framework for understanding individual differences in such attitudes [3, 4]. Early family experiences shape internal working models that guide expectations about closeness, trust, and dependence in later relationships. Yet these processes are embedded in sociocultural contexts. A longstanding debate concerns whether family processes (e.g., chronic, unresolved interparental conflict) or family structure (e.g., parental divorce) matters more for offspring adjustment. Evidence frequently indicates that persistent conflict can be at least as detrimental as, or more consequential than, divorce because it undermines emotional security [5, 6]. This question is especially salient in China, where strong social norms to maintain family integrity may inadvertently prolong children’s exposure to discord.

Recent work with Chinese samples underscores these dynamics. For instance, interparental conflict has been linked to poorer romantic relationship quality among young adults partly through insecure attachment, and attachment avoidance is reliably associated with less favorable relationship evaluations in conflictual contexts [7, 8]. Nevertheless, two gaps remain. First, within Chinese cultural settings, the psychological toll of growing up in high-conflict but intact families has seldom been directly compared with that of divorced families when predicting marital attitudes. Second, although meaning in life is often discussed as a broad protective resource [9], its theoretical limits—whether it actually explains (mediates) or buffers (moderates) the well-documented links between insecure attachment and marital attitudes—are rarely tested.

The present study addresses these gaps in a sample of Chinese university students. We distinguish family process from family structure by comparing emerging adults from harmonious intact families, high-conflict intact families, and divorced families. Guided by attachment theory and prior evidence, we hypothesized that: (H1) attachment avoidance would be negatively associated with more positive marital attitudes; (H2) youth from high-conflict families would report less positive marital attitudes than those from harmonious families; and (H3) differences between divorced and harmonious families would be smaller or nonsignificant. We also conducted an a priori boundary test of meaning in life (H4): we expected concurrent positive correlations with marital attitudes but did not assume that meaning in life would mediate or moderate the association between avoidance and marital attitudes. By clarifying these associations, the study adds culturally relevant evidence to the debate over when interparental conflict matters more than divorce for young people’s views of marriage.

## Methods

### Participants and procedure

We recruited 398 Chinese university students (40.6% male; M age = 20.8 years) who were ≥ 18 and able to complete a Chinese-language questionnaire. Exclusion criteria were declined consent or nonresponse on key variables; missing data were handled via listwise deletion (see Analytic plan). Students were recruited through course announcements and online postings, and completed the survey in supervised classroom sessions using a secure online form during [March–July, 2022]; no financial incentives were provided. After providing electronic informed consent, participants completed measures of parental marital status/conflict, adult attachment, meaning in life, and marital attitudes. The study was approved by the Peking University Medical Ethics Committee (Approval No. 2022126) and conducted in accordance with the Declaration of Helsinki.

### Measures and scoring

#### Parental marital status/conflict (before age 12)

A single categorical item asked participants to select one of four categories: (1) harmonious intact marriage, (2) high conflict but intact, (3) divorced, (4) bereaved. Participants self-categorized based on recalled frequency and intensity of parental arguments before age 12. This distinction follows the family process vs. structure perspective, which emphasizes that sustained interparental conflict can be as consequential as, or more consequential than, divorce for offspring adjustment [5, 10]. For inferential tests, planned contrasts compared high conflict vs. harmonious and divorced vs. harmonious; the bereaved group was summarized descriptively due to small cell size.

#### Adult attachment (ECR)

Attachment avoidance and anxiety were assessed within the Experiences in Close Relationships framework [11, 12]. Items were rated on 5-point Likert scales (1–5) as in the Chinese questionnaire used in this study. Subscale scores were computed per instrument keying (reverse-scored items were recoded prior to aggregation). Higher scores indicate higher avoidance/anxiety. Internal consistencies (Cronbach’s α) appear in Table 1.

#### Meaning in Life (MLQ)

Meaning in life was assessed with the 10-item Meaning in Life Questionnaire [13]. The measure comprises two 5-item subscales-Presence (Items 2, 4, 7, 8, 9) and Search (Items 1, 3, 5, 6, 10)—rated on 7-point scales (1 = strongly disagree to 7 = strongly agree). Following our analytic plan, we (a) reported descriptive statistics for the MLQ total score (sum across 10 items; range = 10–70; higher = stronger meaning) to facilitate comparison with prior work, and (b) used the Presence subscale as the predictor in regression and moderation/mediation tests, given its closer theoretical linkage with well-being and relationship attitudes in the literature [9]. Internal consistency (Cronbach’s α) for the total and subscales is provided in Table 1. Descriptive statistics for the MLQ total are reported to facilitate comparison with prior work, whereas regression/moderation used the MLQ–Presence subscale (see Supplementary Table S1 for subscale descriptives).

#### Marital attitudes

Marital attitudes were assessed with the Marital Attitudes Scale (MAS). We used a 23-item Chinese adaptation validated in college samples [14, 15] (see Supplementary Materials). Items were rated on a 4-point scale (1 = *strongly agree* to 4 = *strongly disagree*). Eight items (1, 3, 5, 8, 12, 16, 19, 23) were reverse-scored, and a total score was computed (range = 23–92), with higher scores indicating more positive attitudes toward marriage. Cronbach’s α for the total score appears in Table 1. Full item wording and keying are provided in the Supplementary Materials.

#### Covariates

Analyses adjusted for gender (male = 1) and age (years). Additional descriptive variables (e.g., bereavement status) were summarized where relevant.

### Analytic plan

We emphasized estimation and transparency consistent with current recommendations.

#### Data screening

We inspected missingness (all key variables < 5%), univariate distributions, and influential cases. Main analyses used listwise deletion; robustness checks excluding cases with |*z*| ≥ 3 produced identical conclusions (Supplementary Materials).

#### Descriptives and correlations

We report means, standard deviations, Cronbach’s α, and Pearson correlations among study variables (Table 1).

#### Group comparisons

One-way ANOVA compared marital attitudes across parental categories with planned contrasts: high conflict vs. harmonious and divorced vs. harmonious. We report partial η² and adjusted pairwise tests where appropriate. The bereaved group was not included in inferential tests due to small *n*.

#### Hierarchical regression

We predicted marital attitudes using stepwise models: Model 1: gender, age, and parental status dummies (reference = harmonious; analytic *N* = 395); Model 2: + attachment avoidance and anxiety; Model 3: + meaning in life (MLQ Presence); Model 4: + Avoidance × Presence interaction. We report standardized β, SE, *p*, and *R²/ΔR²*.

#### Analytic N

Because three respondents did not report parental marital status/conflict, models that include parental status dummies use an analytic sample of *N* = 395; all other analyses use *N* = 398.

#### Mediation and moderation (boundary test)

We tested whether meaning in life mediates or moderates the association between attachment avoidance and marital attitudes. Indirect effects were estimated with bias-corrected bootstrap 95% confidence intervals (5,000 resamples); moderation used centered interaction terms with simple-slope probes. Following best practices, 95% CI excluding zero was required to infer mediation [5, 13].

#### Sensitivity analyses

Appendix reports (a) alternative contrasts (any non-harmonious vs. harmonious; high conflict vs. divorced) and (b) robustness checks to distributional assumptions (e.g., robust regression). Conclusions were unchanged across these specifications.

Analyses were conducted in SPSS/R (version details in Appendix). Two-tailed α = 0.05.

## Results

### Descriptive statistics and correlations

Table 1 presents descriptive statistics, reliability coefficients, and zero-order correlations among the main study variables (*N* = 398). As expected, attachment avoidance was negatively associated with marital attitudes (*r* = − .34, *p* < .001) and meaning in life (*r* = − .16, *p* < .01). Meaning in life was positively associated with marital attitudes (*r* = .20, *p* < .01). Gender differences were also observed: men reported higher marital attitudes than women (*M* = 57.66 vs. 52.77), *F*(1, 396) = 37.33, *p* < .001, partial η² = 0.086 (see Table 2).

### Group differences by parental marital status

A one-way ANOVA tested whether parental marital status was related to marital attitudes (analytic *N* = 395 due to three missing responses). Results indicated a significant effect, *F*(2, 392) = 7.55, *p* < .001, partial η² = 0.054 (see Table 3). Tukey post hoc tests showed that participants from high-conflict intact families reported significantly lower marital attitudes (M = 50.04, SD = 9.57) than those from harmonious intact families (M = 55.56, SD = 7.72; *p* < .001). No significant difference was observed between divorced (M = 54.54, SD = 7.67) and harmonious intact families (*p* = .54).

### Hierarchical regression analyses

To further examine predictors of marital attitudes, we conducted a four-step hierarchical regression (see Table 4). In Step 1, gender and parental marital status (dummy-coded with harmonious intact as the reference) were entered as controls. Men reported more positive marital attitudes (*B* = 2.15, β = 0.18, *p* = .001), and participants from high-conflict intact families reported lower attitudes than those from harmonious intact families (*B* = − 5.52, β = −0.21, *p* < .001). The difference between divorced and harmonious intact families was not significant (*B* = − 1.02, β = −0.03, *p* = .543).

In Step 2, attachment avoidance was added and negatively predicted marital attitudes (*B* = − 0.48, β = −0.34, *p* < .001), accounting for an additional 11% of variance (Δ*R*² = 0.11, *F*change = 47.30, *p* < .001).

In Step 3, meaning in life (Presence) was added and emerged as a significant positive predictor (*B* = 0.22, β = 0.20, *p* = .001), explaining an additional 4% of variance (Δ*R*² = 0.04, *F*change = 10.25, *p* = .001).

In Step 4, the Avoidance × Meaning (Presence) interaction was entered but was not significant (*B* = − 0.12, β = −0.012, *p* = .269; Δ*R*² = 0.002, n.s.), indicating no moderation effect.

As a robustness check, region of origin (urban vs. rural) was included as an additional control; attachment avoidance remained significant (β = −0.301, *p* < .001). Variance inflation factors (all < 10) and tolerance values (> 0.10) indicated no multicollinearity concerns, supporting the robustness of the main findings.

Overall, these results indicate that gender, interparental conflict, and attachment avoidance are significant correlates of emerging adults’ marital attitudes, whereas meaning in life shows a positive direct association but does not function as a mediator or moderator.

**Table 1:** Descriptive statistics, reliabilities, and intercorrelations among study variables (*N* = 398)

Variable	M	SD	1	2	3	4
1. Attachment Anxiety	2.85	0.85	(0.85)			
2. Attachment Avoidance	1.76	0.51	0.35***	(0.77)		
3. Marital Attitudes	54.73	8.17	–0.17**	–0.34***	(0.74)	
4. Meaning in Life	47.30	10.64	0.02	–0.16**	0.20**	(0.91)

The MLQ total score is reported descriptively; regression/moderation analyses used the MLQ–Presence subscale (see Supplementary Materials, Table S1, for subscale descriptives)

Values on the diagonal are Cronbach’s α. All coefficients are Pearson’s r. *p < .05, **p < .01, ***p < .001

**Table 2:** Gender differences in study variables (*N* = 398)

Variable	Male (*n* = 160) M (SD)	Female (*n* = 238) M (SD)	F(1,396)	*p*	partial η²
Attachment Anxiety	2.94 (0.89)	2.79 (0.82)	2.86	0.092	0.007
Attachment Avoidance	1.74 (0.46)	1.77 (0.54)	0.39	0.531	0.001
Marital Attitudes	57.66 (8.03)	52.77 (7.68)	37.33	< 0.001	0.086
Meaning in Life	47.29 (11.87)	47.32 (9.76)	0.00	0.980	0.000

Values are means with standard deviations in parentheses. Effect sizes are reported as partial η²

**Table 3:** Marital attitudes and attachment by parental marital status (*N* = 395)

Variable	Group	*n*	M (SD)	F(2,392)	*p*	partial η²
Attachment Anxiety	Harmonious	312	2.80 (0.84)	2.28	0.079	0.017
	High-conflict intact	55	3.11 (0.82)			
	Divorced	28	2.94 (1.01)			
Attachment Avoidance	Harmonious	312	1.73 (0.51)	4.81**	0.003	0.035
	High-conflict intact	55	1.98 (0.51)			
	Divorced	28	1.65 (0.46)			
Marital Attitudes	Harmonious	312	55.56 (7.72)	7.55***	< 0.001	0.054
	High-conflict intact	55	50.04 (9.57)			
	Divorced	28	54.54 (7.67)			
Meaning in Life	Harmonious	312	47.80 (10.68)	1.17	0.323	0.009
	High-conflict intact	55	45.51 (10.92)			
	Divorced	28	45.14 (9.61)			

Values are means with standard deviations in parentheses. Partial η² = effect size

**Table 4:** Hierarchical regression and mediation/moderation analyses predicting marital attitudes (*N* = 395)

Predictor	B	β	SE	t	*p*	*R*² / ΔR²	F change	95% CI (LL, UL)
Step 1 (Controls)						*R²* = 0.05***		
Gender (0 = female, 1 = male)	2.15	0.18	0.65	3.31	0.001			[0.88, 3.42]
High-conflict intact (vs. harmonious intact)	–5.52	–0.21	1.28	–4.31	< 0.001			[–8.04, − 2.99]
Divorced (vs. harmonious intact)	–1.02	–0.03	1.67	–0.61	0.543			[–4.30, 2.26]
Step 2						*ΔR²* = 0.11***	47.30***	
Attachment avoidance	–0.48	–0.34	0.07	–6.88	< 0.001			[–0.62, − 0.34]
Step 3						*ΔR²* = 0.04**	10.25**	
Meaning in life (presence)	0.22	0.20	0.07	3.21	0.001			[0.08, 0.36]
Step 4						*ΔR²* = 0.002	1.23	
Avoidance × Meaning	–0.12	–0.012	0.11	–1.11	0.269			[–0.34, 0.10]
Indirect effect (PROCESS, 5,000 bootstraps)	–0.022	–	0.14	–	n.s.			[–0.303, 0.260]

Indirect effect estimated using PROCESS (Model 4; 5,000 bootstrap samples)

B = unstandardized coefficient; β = standardized coefficient; CI = confidence interval; LL = lower limit; UL = upper limit; n.s. = not significant. ΔR² = variance explained at each step. Parental marital status dummy-coded with harmonious intact as reference

## Discussion

### Review of findings

This study yields two central insights. First, the quality of parental relationships—specifically, chronic interparental conflict—was more strongly associated with emerging adults’ marital attitudes than family structure (intact vs. divorced). Participants from high-conflict intact families reported less positive marital attitudes than those from harmonious intact families, whereas no difference emerged between divorced and harmonious families (bereavement was described but not analyzed due to small *n*). This pattern underscores that the emotional climate of the family may weigh more heavily than structural continuity in their links to relational orientations.

Second, attachment avoidance showed a robust negative association with marital attitudes, remaining significant after adjusting for gender, parental marital status, and region of origin. By contrast, meaning in life—operationalized as Presence—displayed a positive concurrent link with attitudes but did not mediate or moderate the avoidance–attitude association. Together, these results highlight the salience of interparental conflict and attachment avoidance, and delineate the boundary conditions under which meaning in life contributes to relational cognitions.

### Theoretical implications

These findings align with the Emotional Security Hypothesis and related cognitive–contextual accounts, which emphasize how persistent, unresolved parental conflict undermines children’s sense of safety and fosters maladaptive schemas about intimacy [5, 16]. Consistent with evidence suggesting that conflict can be at least as consequential as divorce, conflictual intact families may be more tightly linked to negative relational cognitions than divorce per se [17]. This supports a growing view that relational quality often matters more than structural status for developmental correlates.

Our results also extend insights from the Family Stress Model [18] by underscoring that relational hostility—independent of structural status—relates to young people’s marital cognitions. Moreover, attachment avoidance appears to be a link between early family experiences and later relational orientations [18], informing theory by showing that avoidance is linked not only to ongoing relationship difficulties but also to anticipatory attitudes toward marriage.

Furthermore, our findings can be interpreted through the lens of Life History Theory. According to this framework, developmental environments function as cues that guide individuals to adopt specific reproductive strategies [19]. Harsh or unpredictable environments—such as those characterized by chronic interparental conflict—may prompt individuals to adopt ‘fast’ life history strategies, which are often associated with earlier sexual maturation and a skepticism toward long-term pair bonding [20]. From this perspective, the negative marital attitudes observed among youth from high-conflict homes may represent an adaptive response to cues of environmental instability, leading them to prioritize self-protection or short-term goals over long-term marital commitments.

### Cultural context

A cultural lens helps interpret these associations. In China, where marital endurance and family reputation are highly valued, some parents may remain married despite chronic conflict [1, 21]. Prolonged exposure to discord may sustain threatening family climates rather than provide the closure that sometimes follows divorce. Emerging evidence with Chinese samples similarly links interparental conflict to lower attachment security and less favorable marital expectations [2, 6]. Our results add cross-cultural evidence that in collectivistic contexts, the quality of marital stability may matter more than stability itself.

Cultural norms of emotional restraint and harmony maintenance may further reinforce avoidant tendencies [22]. Youth exposed to conflict may come to view intimacy as risky, which may be reflected in defensive or skeptical views of marriage—consistent with the observed avoidance–attitude link.

### Boundary conditions of meaning in life

The null mediation and moderation for Presence are theoretically informative. Prior work suggests that existential resources can be domain-specific rather than uniformly protective [23]. For Chinese university students, life meaning may be tied more closely to academic or career goals than to marital expectations [24]. Additionally, attachment operates through relatively automatic, schema-driven processes, whereas meaning reflects higher-order, reflective beliefs [18]; such beliefs may lack potency to override entrenched attachment-based expectations unless expressly targeted. Interventions predicated on meaning-making may therefore need relationship-specific tailoring to influence marital attitudes [25].

### Gender differences in a transforming China

Men reported more positive marital attitudes than women. This should be interpreted within rapid sociocultural change: marriage registration has declined, and public discourse highlights rising costs, rigid gender roles, and parenting burdens [26, 27]. Women’s disproportionate opportunity costs under limited work–family support may foster greater skepticism toward marriage. Similar patterns across East Asia—amid expanding female education and employment but incomplete institutional accommodation—suggest that the gender difference likely reflects adaptive responses to structural constraints [28, 29].

### Practical and policy implications

Family policy and intervention. Efforts should prioritize reducing children’s exposure to chronic interparental hostility and promoting constructive conflict resolution rather than focusing solely on preventing divorce.

#### Higher education

Campus counseling and psychoeducation can address family-of-origin influences and help students identify and revise avoidant schemas.

#### Clinical practice

For clients high in avoidance, meaning-focused work alone may be insufficient; emotion-focused, trust-building, and intimacy-focused approaches may be needed alongside reflective meaning-making.

### Limitations and future directions

Several limitations warrant caution. First, parental conflict was assessed categorically via a single item, which simplifies complex dynamics; future work should use validated dimensional measures and multi-informant designs. Second, the cross-sectional, self-report design precludes causal inference; longitudinal and multi-wave studies are needed. Third, the university sample limits generalizability; replication with diverse age, socioeconomic, and regional groups is warranted.

Finally, although Presence correlated positively with marital attitudes, it neither mediated nor moderated the avoidance–attitude link. This null result may reflect measurement choices (using Presence rather than a composite or relationship-specific meaning), statistical power for small indirect effects, or cultural factors tethering meaning to non-relational domains. Future work should test alternative mechanisms (e.g., trust, fear of intimacy, emotional deactivation) and conduct cross-cultural comparisons to clarify when meaning’s protective role emerges.

### Conclusion

Interparental conflict and attachment avoidance show robust associations with marital attitudes among Chinese emerging adults. By demonstrating that hostile intact families are more closely linked to negative marital attitudes than divorce per se—and that avoidance relates to less favorable attitudes independently of demographics—this study advances theory and practice. It challenges purely structural explanations of family associations, underscores the durability of attachment pathways, and clarifies the boundary conditions of meaning in life. Theoretically, the work integrates emotional security and cognitive–contextual perspectives with attachment theory in a collectivistic context; practically, it suggests that fostering emotional security and addressing avoidance are promising targets for supporting healthier intimate relationships in emerging adulthood.These considerations are consistent with global syntheses indicating that the quality of interparental interaction, rather than marital status per se, is more predictive of offspring adjustment [30].

## Supplementary Information

Supplementary Material 1.

## Data Availability

Anonymized data and analysis scripts will be deposited in an open repository upon acceptance; the persistent link (DOI) will be provided in the published article. Until then, the materials are available from the corresponding author on reasonable request.

## Abbreviations

ECR: Experiences in Close Relationships
MLQ: Meaning in Life Questionnaire
MAS: Marital Attitudes Scale
ANOVA: Analysis of Variance
SD: Standard Deviation
CI: Confidence Interval
