# Supplementary material for: Interparental conflict, not divorce, is linked to less positive marital attitudes among Chinese emerging adults
Source: BMC Psychol. 2026 Feb 24;14:437. doi: 10.1186/s40359-026-04232-y (PMC13036988; doi:10.1186/s40359-026-04232-y)
Supplement: Supplementary file 1 — Supplementary Material 1. [file 40359_2026_4232_MOESM1_ESM.docx]

# Supplementary Materials

## S1. Measures: full wording and scoring details

**S1.1 Marital Attitudes Scale (MAS): Chinese adaptation**

Items & response format: 23 items, 4-point scale (1 = strongly agree to 4 = strongly disagree).

Reverse-keyed items (8): 1, 3, 5, 8, 12, 16, 19, 23.

Scoring: Reverse the 8 keyed items, then sum all 23 items to a total score (range 23–92); higher = more positive attitudes toward marriage.

Reference chain: Original MAS: Braaten & Rosén (1998) [13]; Chinese adaptation: Zhang & Sun (2017) [14].

**S1.2 Meaning in Life Questionnaire (MLQ)**

Structure: 10 items, two 5-item subscales (Presence; Search), 7-point response scale (1–7).

Subscale mapping: Presence (items 2, 4, 7, 8, 9); Search (items 1, 3, 5, 6, 10).

Scoring used in this study: Descriptives are reported for the MLQ total (10–70); regression/moderation used the Presence subscale.

**S1.3 Adult attachment (ECR framework)**

Subscales: Avoidance and Anxiety on 5-point scales; scored per instrument keying (reverse as required), then compute subscale totals/means.

## S2. Data screening, missingness, and exclusions

Inclusion criteria: university students aged ≥18; able to complete a Chinese-language questionnaire.

Exclusion criteria: declined consent; nonresponse on key variables; missing data handled via listwise deletion in main analyses.

Missingness: all key variables <5%; parental marital status/conflict had 3 missing responses (analytic N = 395 for that ANOVA).

Outliers: sensitivity analyses excluding cases with |z| ≥ 3 yielded unchanged conclusions (see S4).

Final analytic Ns: correlations/regressions, N = 398; group comparisons by parental status/conflict, N = 395.

Administration window: [March–July, 2022]; supervised classroom sessions; no incentives.

## S3. Additional robustness checks

### S3.1 Pairwise Welch’s t-tests for group differences in marital attitudes

To address potential heterogeneity of variances across parental categories, we conducted heteroscedastic (Welch) pairwise t-tests using the summary statistics reported in the main text (Table 3). Results corroborated the one-way ANOVA pattern: participants from **high-conflict** intact families reported significantly **lower** marital attitudes than those from **harmonious** intact families, whereas **divorced** and harmonious families did not differ.

**Contrast statistics (Welch method, two-tailed):**

| **Contrast** | **Mean difference ΔM (Group1 − Group2)** | **t** | **df (Welch)** | **p** | **95% CI for ΔM** |
| --- | --- | --- | --- | --- | --- |
| High conflict vs Harmonious | **−5.52** | **−4.052** | **66.9** | **0.0001** | **[−8.24, −2.80]** |
| Divorced vs Harmonious | **−1.02** | **−0.674** | **32.1** | **0.5053** | **[−4.10, 2.06]** |

Notes. ΔM is computed as **Group1 − Group2** using group means and SDs from the main text (Harmonious: n = 312, M = 55.56, SD = 7.72; High conflict: n = 55, M = 50.04, SD = 9.57; Divorced: n = 28, M = 54.54, SD = 7.67). Welch degrees of freedom are Satterthwaite-adjusted. Confidence intervals refer to the difference in means.

### S3.2 Effect sizes (Hedges’ g) for key contrasts

We computed bias-corrected standardized mean differences (Hedges’ g) from the reported means, SDs, and Ns:

| Contrast | Hedges’ g | 95% CI (LL, UL) | Interpretation |
| --- | --- | --- | --- |
| High conflict – Harmonious | -0.687 | [-0.977, -0.396] | medium |
| Divorced – Harmonious | -0.132 | [-0.518, 0.254] | negligible (n.s.) |

## S4. Mediation and moderation specifications

Mediation tested with PROCESS Model 4 (bias-corrected bootstrap, 5,000 resamples); moderation via centered interaction terms with simple-slope probes at ±1 SD. The Avoidance × Presence interaction was not significant (b = −0.12, SE = 0.11, p = .269). The indirect (Avoidance → Presence → Marital attitudes) 95% CI included zero (n.s.).

## S5. Reliability complements

Cronbach’s α values are reported in the main Table 1. 95% CIs can be provided upon request.

## S6. Alternative contrasts and sensitivity models

Alternative contrasts (any non-harmonious vs harmonious; high conflict vs divorced) and outlier-robust re-estimations yielded unchanged conclusions compared to the main analysis.

## S7. Ethics and data availability

Ethics approval: Peking University Medical Ethics Committee (Approval No. 2022126). Conducted in accordance with the Declaration of Helsinki.

Informed consent: Electronic informed consent obtained from all participants.

Compensation: None.

Data/code availability: Upon acceptance, anonymized data and scripts will be shared in an open repository (URL/DOI to be provided).
